# Effects of Fermentation with *Eurotium cristatum* on Sensory Properties and Flavor Compounds of Mulberry Leaf Tea

**DOI:** 10.3390/foods13152347

**Published:** 2024-07-25

**Authors:** Xiaoyu Yang, Zijun Liu, Yanhao Zhang, Shuangzhi Zhao, Shigan Yan, Liping Zhu, Qingxin Zhou, Leilei Chen

**Affiliations:** 1 Institute of Food and Nutrition Sciences and Technology, Shandong Academy of Agricultural Sciences, Jinan 250100, China; yuren04.1@hotmail.com (X.Y.); zhang_8497@163.com (Y.Z.); zhaoshz717@163.com (S.Z.); nkychenleilei@shandong.cn (L.C.); 2 School of Bioengineering, Qilu University of Technology, Shandong Academy of Sciences, Jinan 250353, China; 18954171550@163.com (Z.L.); yanshigan@126.com (S.Y.)

**Keywords:** Mulberry leaf tea fermentation, *Eurotium cristatum*, taste property, relative odor activity value (ROAV), key aroma-active compound, flavor characteristic

## Abstract

Mulberry leaf tea (MT) is a popular Chinese food with nutrition and medicinal functions. Solid-state fermentation with *Eurotium cristatum* of MT (FMT) can improve their quality. Differences in chromaticity, taste properties, and flavor characteristics were analyzed to evaluate the improvements of the sensory quality of FMT. After fermentation, the color of the tea infusion changed. The E-tongue evaluation results showed a significant decrease in unpleasant taste properties such as sourness, bitterness, astringency, and aftertaste-bitterness, while umami and saltiness taste properties were enhanced post-fermentation. Aroma-active compounds in MT and FMT were identified and characterized. A total of 25 key aroma-active compounds were screened in MT, and 2-pentylfuran showed the highest relative odor activity value (ROAV). A total of 26 key aroma-active compounds were identified in FMT, and the newly formed compound 1-octen-3-one showed the highest ROAV, which contributed to FMT’s unique mushroom, herbal, and earthy flavor attributes. 1-octen-3-one, (E)-2-nonenal, trimethyl-pyrazine, 2-pentylfuran, and heptanal were screened as the potential markers that contributed to flavor differences between MT and FMT. *E. cristatum* fermentation significantly altered the sensory properties and flavor compounds of MT. This study provides valuable insights into the sensory qualities of MT and FMT, offering a theoretical basis for the development of FMT products.

## 1. Introduction

Mulberry leaves are a functional food, as well as traditional Chinese medicine [1]. The leaves or their extracts have pharmacological effects, such as hypoglycemic effects, hypolipidemic effects, antioxidant effects, anti-inflammatory effects, anticancer, and so on [2,3]. The medicinal value of mulberry leaves lies in their multiple bioactive ingredients [2,4]. Because of their various bioactive ingredients and pharmacological activities, mulberry leaves are made into mulberry leaf tea (MT) and are accepted by the public. Processing MT can increase its active ingredient content, enhance its function, and change its sensory characteristics. Fermentation is a widely used food processing method. Mulberry leaves fermentation by *Ganoderma lucidum* can improve the content of 1-deoxynojirimycin to 0.402%, which is 1.2-fold of unfermented mulberry leaves [5]. Fermentation with different microbes of mulberry leaves increased the total phenolics and flavonoids contents, which, in turn, enhanced the antioxidant activities and hypoglycemic activities [6,7]. Therefore, the fermentation of MT with microbes is an effective approach to improve its qualities.

*Eurotium cristatum* is a kind of probiotic fungus isolated from Chinese traditional post-fermented tea [8,9,10]. *E. cristatum* secretes extracellular enzymes to promote a series of complex chemical reactions such as oxidation, condensation, and polymerization of ingredients in substances [10]. Nowadays, it has been widely used in the fields of tea, beans and grains processing, and deep processing of traditional Chinese medicine. Fermentation with *E. cristatum* gave the substrates unique sensory qualities and enhanced their bioactivities [11,12,13,14]. Several studies have focused on *E. cristatum* fermentation of mulberry leaves. After solid-state fermentation of mulberry leaves with *E. cristatum*, the release of total flavonoids was enhanced, and 2,2′-azinobis-(3-ethylbenzthiazoline-6-sulphonate) (ABTS) radical scavenging activities and α-glucosidase inhibitory activities were increased [15]. Yang et al. produced a kind of mulberry leaf Fu loose tea via *E. cristatum* solid-state fermentation of mulberry leaf raw dark green tea. The mulberry leaf Fu loose tea showed higher contents of tea pigments and decreased contents of polyphenols, flavonoids, and polysaccharides. The fermented tea exhibited better α-glucosidase inhibitory activity [16]. However, as a potential new tea product with health benefits, there is no research on the flavor characteristics and aroma substances of fermented mulberry leaf tea, which plays an important role in the evolution of tea qualities.

The unique flavors, aroma compounds, and transformation mechanisms have been researched using multi-omics. The odor-active compounds of different aroma types (FBT) were detected by HS-SPME combined with GC-MSO [17]. The influence of pile-fermentation on the aroma characteristic of dark tea was studied by GC×GC-QTOFMS combined with E-nose and GC-O techniques. The aroma compounds such as naphthalene, 2-methylnaphthalene, and dibenzofuran increased the woody flavor; at the same time, (Z)-4-heptenal, 2-nonenal, and 1-hexanol enhanced the mushroom, fatty, and sweet flavors [18]. Microbial fermentation imparted the unique flavor of Pu-erh tea, as the volatile compounds were detected by the GC-MS technique. The odor activity value was used to investigate the key aroma-active compounds and the flavor attributes of Pu-erh tea [19,20,21].

In this study, fermented mulberry leaf tea (FMT) was made by *E. cristatum* solid-state fermentation of mulberry leaf tea, and the sensory qualities of MT and FMT were systematically analyzed in this study. The differences in taste and color properties of tea infusions between MT and FMT were analyzed by electronic tongue and colorimeter. Furthermore, the key aroma compounds and flavor characteristics of MT and FMT were further investigated by combining GC×GC-TOF MS with the relative odor activity value (ROAV). This research objectively evaluated the changes in sensory qualities of FMT, which is helpful in the development of FMT products.

## 2. Materials and Methods

### 2.1. Materials and Chemicals

MT (made from mulberry leaves) was the commercially available product purchased from Bozhou City Wenshun Pharmaceutical Co., Ltd. (Bozhou, China). *E. cristatum* was previously obtained from fermented tea in our laboratory after screening and optimization of a series of conditions, with fast growth rate and strong fermentation ability, named NCPS2018015, which was identified as *E. cristatum*. The strain was stored in the China Center for Type Culture Collection (CCTCC, Wuhan University, Wuhan, China) with the conservation number CCTCC NO: M2018882. Malt extract medium was purchased from Sinopharm Chemical Reagent Co., Ltd. (Shanghai, China). n-hexyl-d13 was obtained from C/D/N Isotopes Inc. (Pointe-Claire, QC, Canada). Ethanol was purchased from Shanghai Aladdin Biochemical Technology Co., Ltd. (Shanghai, China). N-alkane was purchased from Sigma, St. Louis, MO, USA. N-hexane was purchased from Yonghua Chemical Co., Ltd. (Shanghai, China). A solid-phase microextraction fiber coated with divinylbenzene/carboxen/polydimethylsiloxane (DVB/CAR/PDMS, 50/30 μm × 1 cm) assembly was obtained from Supelco (Bellefonte, PA, USA).

### 2.2. Solid-State Fermentation

*E. cristatum* was activated for two generations on malt extract medium plates. *E. cristatum* plates were incubated for 7 days at 28 °C, and *E. cristatum* formed a large number of golden spores on the surface of the culture medium. Under sterile conditions, golden spores and 100 mL of sterile water were added to a 250 mL triangular flask containing glass beads and then shaken at 28 °C and 220 rpm for 20 min in a thermostatic shaker. Next, the spore suspension was diluted to 10^6^ spores/mL by a hemocytometer counting chamber.

MT (18 g) was mixed with distilled water (12 mL) and sterilized for 1 h at 121 °C. After cooling to room temperature, 1.5 mL of diluted spore suspension was added, and the culture was carried out at a constant temperature (28 °C) for 6 days. FMT was obtained. MT and FMT were quickly frozen with liquid nitrogen, lyophilized, and stored at −80 °C for future use. Each sample was prepared in triplicate.

### 2.3. Electronic Sensory Analysis

First, 2.50 g of the sample was infused with 125 mL of freshly boiled water for 5 min, and then, the tea leaves were filtered out. Before the experiment, the tea infusion was cooled to room temperature (25 °C). The tea infusion was collected for the subsequent electronic sensory analysis.

#### 2.3.1. Chromatic Analysis of Tea Infusion

The chromaticity and brightness of the tea infusion was measured by a WSC-2B portable precision colorimeter (Shanghai INESA Physico-Optical Instrument Co., Ltd., Shanghai, China) [16]. The color parameter of tea infusion, i.e., the L* value, represents the degree of lightness: 100, completely white, and 0, completely black. The a* value indicates redness or greenness: positive value = red, and negative value = green. The b* value indicates yellowness or blueness: positive value = yellow, and negative value = blue. The ΔE* value represents the total variation in lightness and hue. Calibration with standard black and white tiles was performed prior to using the colorimeter. The tea infusion was then placed into a colorimetric dish for detection.

#### 2.3.2. E-Tongue Measurement

The taste quality of MT and FMT was evaluated using Taste-Sensing System SA402B (Intelligent Sensor Technology Co., Ltd., Atsugi, Japan) [22]. The sensor array of E-tongue consists of taste sensors and reference electrodes. Taste sensors include umami, sourness, saltiness, bitterness, and astringency sensors. Not only that, it also analyzed its aftertaste-bitterness, aftertaste-astringency, and richness. The E-tongue measurement was composed of three phases: cleaning phases (5.5 min), sample taste test phases (30 s), and sample aftertaste test phases (30 s). Each sample was prepared in triplicate. The tea infusion was measured four times to obtain an average value.

### 2.4. Determination of Volatile Organic Compounds

#### 2.4.1. Extraction of Volatile Organic Compounds

Headspace solid-phase microextraction (HS-SPME) was used to extract the volatile organic compounds (VOCs) of MT and FMT [23]. Internal standard solution: An appropriate amount of standard substance (n-Hexyl-d13) was dissolved in 50% ethanol aqueous solution to prepare the 1 mg/L single standard mother liquor, which was stored in the refrigerator at 4 °C. At the same time, 1000 mg/L n-alkane standards were diluted step by step with n-hexane to prepare a solution of 1 mg/L and stored in a refrigerator at 4 °C.

Transfer 500 mg of the stored sample to a 20 mL headspace vial. Then, add 4 mL of saturated sodium chloride aqueous solution to the vial. Next, introduce 10 μL of the internal standard (ISTD) solution into each sample. Incubate the samples at 60 °C for 10 min. Before extracting the samples, put the SPME fiber in a chamber set at 270 °C for 10 min. Subsequently, transfer the SPME fiber to an incubator maintained at 60 °C for 40 min. Desorb the SPME fiber by subjecting it to a temperature of 250 °C for 5 min within the GC injector system. After injection, return the SPME fiber back into the chamber set at 270 °C for 10 min. Finally, transfer exactly 10 μL of n-alkanes into another separate headspace vial with a volume capacity of 20 mL. Perform incubation extraction and subsequent injection as required.

#### 2.4.2. GC×GC Analysis

The LECO Pegasus BT 4D (LECO, St. Joseph, MI, USA) GC×GC-TOF MS chromatography system was developed by Agilent 8890, a gas chromatograph (Agilent Technologies, Palo Alto, CA, USA), the two-stage injection modulator, and shunt; the mass spectrometry system was a high-resolution TOF mass spectrometry detector in accordance with the method described by Yang et al. [24]. The separation system comprised a one-dimensional chromatographic column: Rxi-5Sil MS (30 m × 250 μm I.D., 0.25 μm) (Restek, Bellefonte, PA, USA) and two-dimensional chromatographic column Rxi-17Sil MS (2.0 m × 150 μm I.D., 0.15 μm) (Restek, USA). High-purity helium was used as a carrier gas at a constant flow rate of 1.0 mL/min. One-dimensional chromatographic column Rxi-5Sil MS: The initial temperature was 50 °C and held for 1 min at a rate of 2 °C/min 1 min at 170 °C, kept at a speed of 30 °C/min to 230 °C, and kept 1 min. The two-dimensional chromatographic column Rxi-17Sil MS was higher than the temperature program of the one-dimensional chromatography column at 5 °C, the temperature of the modulator was always higher in 2 d when the chromatographic column temperature was 15 °C, and the modulation cycle was 15 s. The injection port temperature was 250 °C.

#### 2.4.3. Mass Spectrum Conditions

For the LECO Pegasus BT 4D mass spectrometer detector (LECO, St. Joseph, MI, USA), the mass spectrometer transmission line temperature was 250 °C, the ion source temperature was 250 °C, the acquisition rate was 200 spectra/s, the electron impact source was 70 eV, and the detector voltage was 2039 V. The mass spectrum was scanned from *m*/*z* 35 to 550.

### 2.5. Data Statistics and Analysis

The column chart and radar image of electronic tongue data were generated using Origin 2021. Chroma TOF 5.55.35 software was used to analyze the raw data of the flavor compounds and annotate them from the NIST2020 database. The discrepancy between the retention index (RI) of the compound analyzed by mass spectra and the reference retention index in the database (Lib-RI) did not exceed 20. PubChem database and Classyfire [25] were used to analyze the category annotation of the detected flavor substances, and the number and relative content of each category of the flavor substances were analyzed. BioDeep platform (https://www.biodeep.cn) was used for visualization of the changes of the VOC profiles of MT and FMT. Odor database and FlavorDB [26] were used to analyze the sensory flavor characteristics of the substances. ROAV was used to assess the contribution of each volatile component to the overall flavor [27]. The formula for ROAV is as follows:

$${ROAV}_{i}=100\times (\frac{{Peak}_{i}}{T_{i}})/(\frac{{Peak}_{A}}{T_{A}}).$$

*Peak_A_* and *T_A_* represent normalized quantitative values and odor values of the compound with the minimum odor threshold, respectively. The ROAV value of the compound with the minimum odor threshold was set to 100. *Peak_i_* and *T_i_* represent normalized quantitative values and odor values of the compound to be measured, respectively [27]. Igraph v1.3.5 and FlavorDB (https://cosylab.iiitd.edu.in/flavordb/, accessed on 21 June 2024) were used to build an association graph of flavor compounds and their sensory characteristics for analysis of the flavor characterization of differential compounds.

## 3. Results and Discussion

### 3.1. Effects of Fermentation on the Color and Taste Qualities of MT and FMT

**Figure 1:**
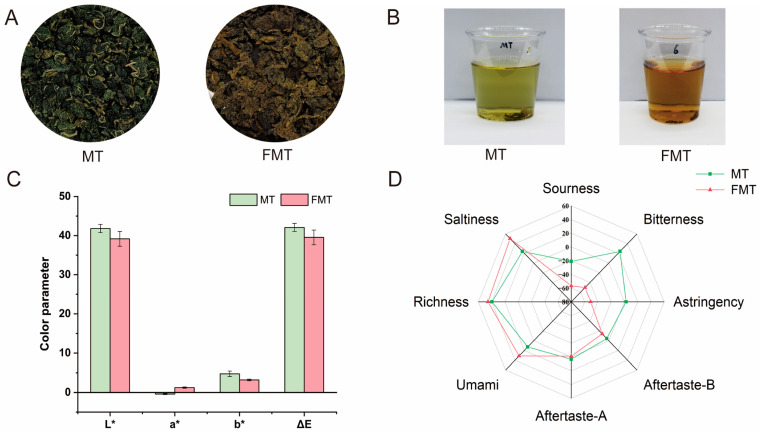
Sensory quality of MT and FMT. (**A**) The appearances of MT and FMT. (**B**) Tea infusion of MT and FMT. (**C**) Color parameter of tea infusion. (**D**) E-tongue analysis of taste characteristics.

In order to evaluate the color and taste properties of MT and FMT in a more objective mode, we used the E-tongue and colorimeter for testing. FMT had the typical characteristics of FBT. The surface of FMT was covered with golden spores of *E. cristatum* (Figure 1A). The color and brightness of the tea infusion could impact consumer choices. The color of the tea deepened with the progress of fermentation, with yellow-green gradually changing to reddish-brown (Figure 1B). As shown in Figure 1C, the L* value of FMT infusion decreased slightly, which represented the brightness of tea infusion decreased after fermentation, and the a* values of FMT infusion were significantly higher than MT infusion, which meant that FMT infusion was redder and MT infusion was greener. The results were consistent with previous reports [16]. The results of the b* value showed that MT infusion was yellower. Theabrownins are the main tea pigments that affect the color of tea infusion. The darker the color of tea infusion, the higher the content of theabrownins [22]. The change in infusion color intuitively indicated the different contents of theabrownins thereof. The fermentation of MT with *E. cristatum* might increase the contents of theabrownins.

The taste properties of tea infusions are shown in Figure 1D. Compared to MT, the signal values of umami and saltiness sensors of FMT were obviously increased, whereas the sourness, astringency, and bitterness signals values of FMT were decreased. Moreover, the aftertaste-bitterness and aftertaste-astringency signals were also reduced after fermentation. Previous studies indicated that the taste properties of tea could reflect its chemical profile [22]. Catechins and alkaloids have been reported as the main contributors to the bitter taste and astringency [28], and theabrownins are negatively correlated with bitter taste. During the fermentation process, catechins in MT were oxidized and polymerized into theabrownins. The contents of theabrownins were increased after fermentation; therefore, the bitterness and astringency decreased [29]. Research also showed that theabrownins exhibited a strong umami taste [30]. Moreover, the salty taste of tea was attributed to free amino acids and tea polysaccharides [22,29]. It is worth noting that FMT had a special fungus fragrance. The changes in the taste properties were caused by the fermentation of *E. cristatum*.

### 3.2. Variation of VOC Profile of MT and FMT

In order to investigate the changes in the aroma components after fermentation, the VOCs of MT and FMT were extracted by HS-SPME and analyzed based on GC×GC-TOF-MS. This method allowed for the detection of a wider range of VOCs [31]. It was found that the signal nature and intensity of MT and FMT varied significantly in the three-dimensional total ion flow chromatogram (Figure S1).

**Figure 2:**
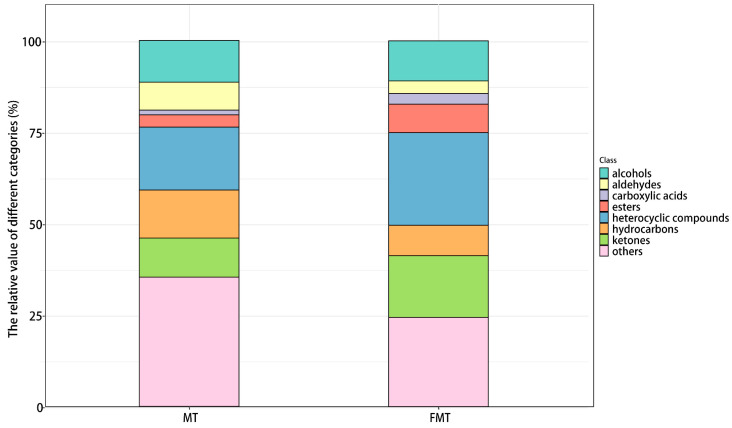
Relative abundance of different types of volatiles.

A total of 1466 VOCs were detected in the MT samples, and 1538 VOCs were detected in the FMT samples. Among them, 812 typical compounds were identified in MT, while 884 typical compounds were identified in FMT. The identified VOCs were classified into eight categories, and the number and relative content of each category were obtained (Figure 2). Heterocyclic compounds were the most abundant category in FMT, with 239 kinds; the others were, in turn, hydrocarbons (218 kinds), ketones (167 kinds), alcohols (100 kinds), esters (70 kinds), aldehydes (45 kinds), carboxylic acids (31 kinds), and other compounds. In MT, seven classes of volatile compounds were identified. Among them, there were 226 types of hydrocarbons, 197 types of heterocyclic compounds, 138 types of ketones, 93 types of alcohols, 83 types of esters, 43 types of aldehydes, 27 types of carboxylic acids, and other compounds. After fermentation, the relative content of aldehydes decreased from 7.66% to 3.41%, and hydrocarbons decreased from 13.14% to 8.37%. Meanwhile, the relative content of carboxylic acids (1.20–2.92%), esters (3.36–7.86%), ketones (10.77–16.76%), and heterocyclic compounds (17.28–25.40%) were significantly increased. These results indicated that fermentation by *E. cristatum* altered the composition of VOCs of MT.

**Figure 3:**
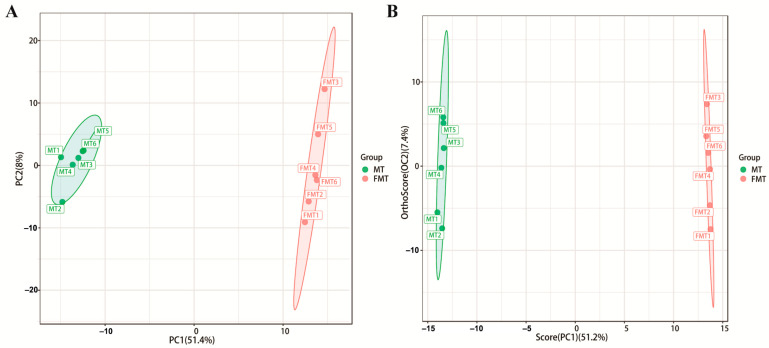
Results of the multivariate statistical analysis of volatile compounds. (**A**) PCA score plot, R2X = 59.4%; (**B**) OPLS-DA score plot, R2X = 58.6%, R2Y = 100%, Q2 = 98.8%. MT1–6 represent 6 parallel samples of MT, and FMT1–6 represent 6 parallel samples of FMT.

Principal component analysis (PCA) was used to obtain the interrelationships between different samples. As shown in Figure 3A, the PCA indicated that the two principal components accounted for 51.6% and 8% of the overall variances in the mode, with a total explained variance of 59.4%. MT and FMT clustered within the group, and the difference in volatile profiles between the groups was significant. Based on the PCA, an orthogonal projections to latent structures discriminant analysis (OPLS-DA) model was constructed to analyze the differences in VOCs of MT and FMT (Figure 3B).

**Figure 4:**
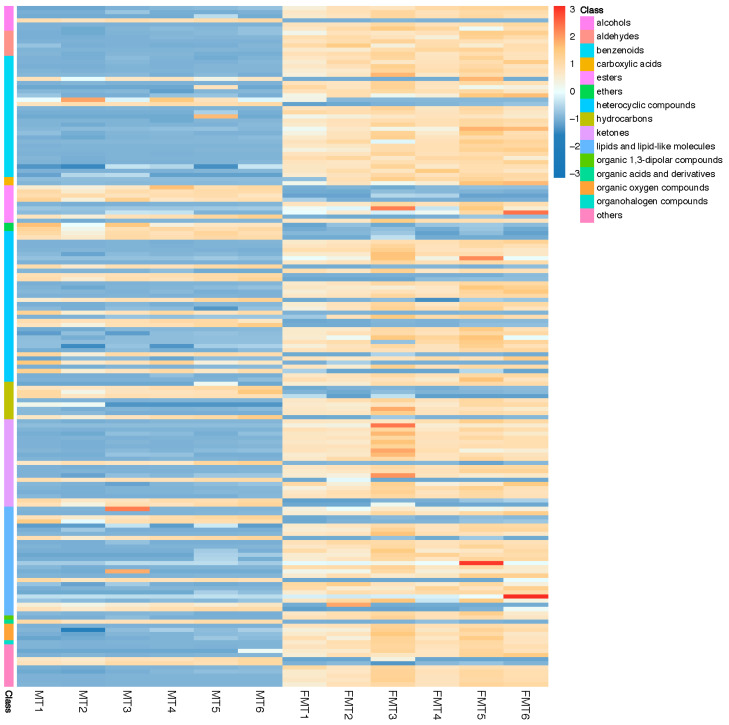
Heatmap analysis of the critical volatile compounds in the tea samples. A color-coded scale from blue to red corresponds to the content of critical metabolites shifting from low to high. MT1–6 represent 6 parallel samples of MT, and FMT1–6 represent 6 parallel samples of FMT.

Based on the OPLS-DA model, the Variable Importance for the Projection (VIP) diagram was obtained. Two conditions were used to select the relevant differential compounds among MT and FMT: *p* < 0.05 and VIP > 1. We screened 200 VOCs as differential compounds, of which 144 were upregulated and 56 were downregulated in FMT compared to MT. To further analyze the differential compounds, heat maps were drawn with fold change (FC) > 2 or FC < 0.5 as the screening conditions to show the differences between the MT and FMT groups (Figure 4). The cluster heat map showed the potential differences and associations among the VOCs in MT and FMT. According to the heat map analysis, the relative contents of most VOCs were upregulated after fermentation.

### 3.3. Effects of Fermentation on the Composition of Aroma-Active Compounds in MT and FMT

The flavor characteristic of tea is determined by the combination of different aroma compounds with different odor characteristics and different aroma contribution degrees [32]. In order to evaluate the contribution of VOCs to the flavor characteristic, the ROAV of the compounds were calculated. ROAV is a measure of the minimum threshold at which humans perceive taste, and the ROAV can be used to assess the contribution of each volatile component to the overall flavor, and larger values of ROAV were considered to contribute more [27]. The compound with ROAV ≥ 1 was considered as a key aroma compound [33]. We used the Odor database and FlavorDB jointly to analyze the odor characteristics of the aroma compounds [26]. These databases related the chemical features of the VOCs to odors and flavors, providing a comprehensive understanding of the flavor attributes. Table 1 summarizes the key aroma compounds in MT and FMT. A total of 33 aroma-active compounds were identified in MT and FMT, including ketones, aldehydes, alcohols, heterocyclic compounds, benzenoids, lipids and lipid-like molecules, and esters. It is worth noting that ketones, alcohols, heterocyclic compounds, lipids, and lipid-like molecules become more abundant after fermentation. The number of aldehydes was highest in the aroma-active compounds of MT, while ketones were the most abundant substances in the aroma-active compounds of FMT. In MT, 2-pentylfuran showed the highest ROAV (10,601.94), followed by heptanal (2878.87) and trimethyl-pyrazine (1311.26). The high ROAV of these compounds indicated that these three compounds might be the most important contributors to the flavor characteristics formation to MT, and there was a total of 25 aroma compounds with ROAV ≥ 1 in MT (Table 1). The compound with the highest ROAV in FMT was 1-octen-3-one (3950.26), while compounds with ROAV ≥ 1000 included trimethyl-pyrazine (3026.34) and (E)-2-nonenal (1756.96), and there were 26 aroma compounds with ROAV ≥ 1 in FMT (Table 1). However, some of the key aroma compounds (octanal, hexanal, and 6-methyl-5-hepten-2-one) of MT were not the key aroma compounds in FMT due to their ROAV < 1 in FMT. This was attributed to the fermentation of *E. cristatum* [30]. In addition to this, trimethyl-pyrazine, (E)-2-octenal, 2-ethylhexanol, acetophenone, isophorone, naphthalene, phenol, and a-terpineol had higher ROAV values in FMT compared to MT, suggesting that these aroma compounds contributed more to the flavor characteristics of FMT than MT. Based on changes in the relative contents of VOCs, OPLS-DA, and ROAV (ROAV > 1000, FC > 2 or <0.5, and VIP > 1) [21], the potential markers that contribute to aroma differences in MT and FMT were screened. 1-octen-3-one, (E)-2-nonenal, trimethyl-pyrazine, 2-pentylfuran, and heptanal were the potential aroma markers between MT and FMT.

### 3.4. Key Aroma-Active Compounds Forming Different Flavor Characteristics of MT and FMT

**Figure 5:**
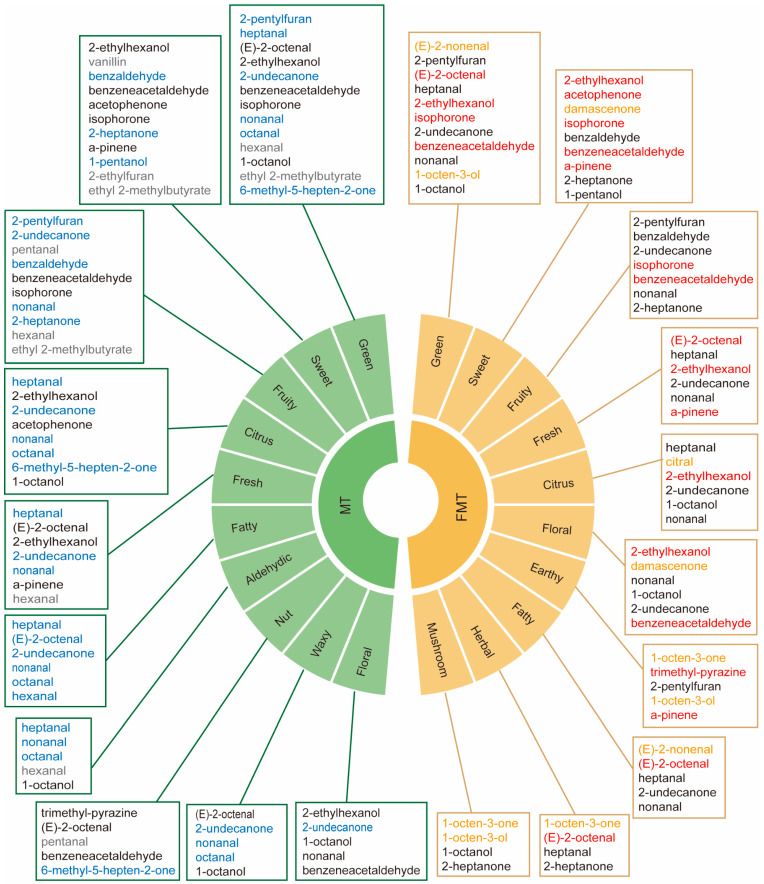
Key aroma-active compounds form the dominant flavor characteristics of MT and FMT. Compounds labeled in blue are downregulated after fermentation, compounds labeled in gray disappeared after fermentation, compounds labeled in red are upregulated after fermentation, and compounds labeled in saffron yellow are newly formed after fermentation.

**Figure 6:**
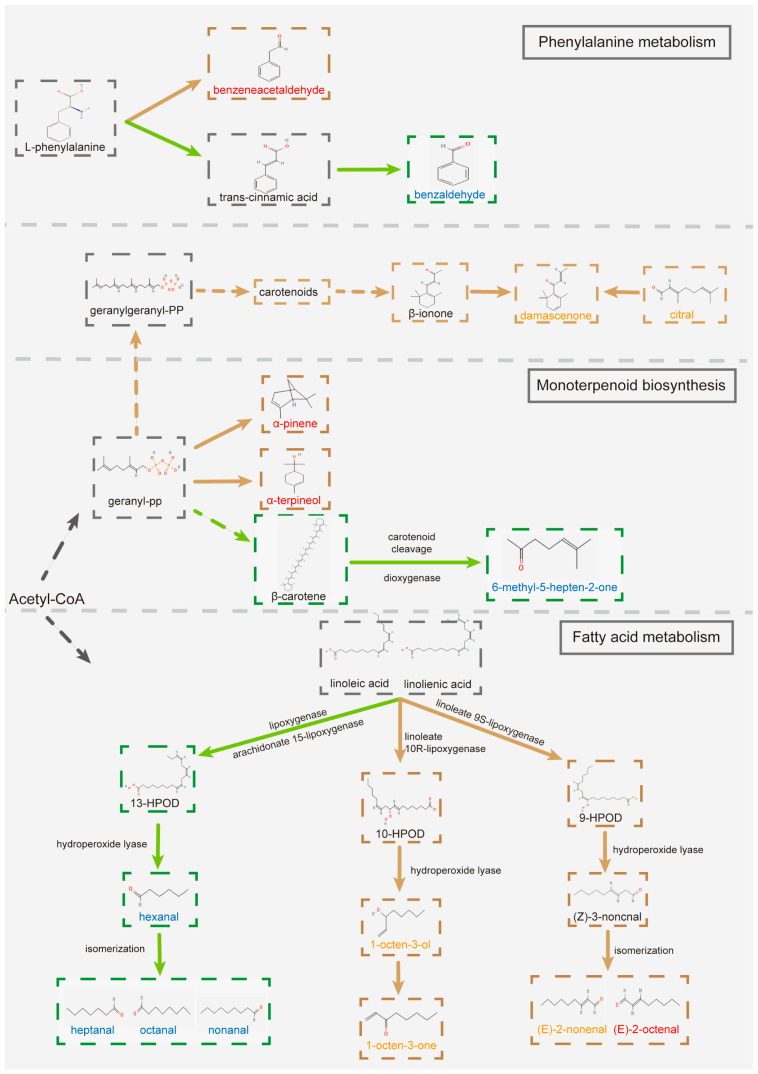
Probable metabolic pathway of the key aroma-active compounds. Compounds labeled in blue are downregulated after fermentation, compounds labeled in red are upregulated after fermentation, and those labeled in saffron yellow are newly formed after fermentation.

According to the flavor characters of key aroma-active compounds and the frequency of each flavor character, we screened out the dominant flavor characteristics of MT and FMT. The flavor properties of MT and FMT are shown in Figure 5. Both MT and FMT had green, sweet, fruity, fresh, citrus, fatty, and floral attributes, while MT had three unique flavor attributes (aldehydic, nut, and waxy), and FMT had the novel earthy, herbal, and mushroom flavor attributes. The key flavor compounds that contributed to the dominant flavor characteristics of MT and FMT are listed in Figure 5. Among these compounds, the upregulated compounds after fermentation were (E)-2-octenal, 2-ethylhexanol, isophorone, benzeneacetaldehyde, a-pinene, acetophenone, and trimethyl-pyrazine. In addition, the new-formed compounds were 1-octen-3-one, 1-octen-3-ol, citral, and (E)-2-nonenal. These upregulated or newly formed substances helped FMT maintain the pleasant sweet, fresh, fruity, and floral flavors while also making “earthy”, “herbal”, and “mushroom” to be the new dominant flavor characteristics of fermented tea. Mushroom aroma attribute has been widely concerning in the research of fermented teas [18,34,35]. 1-octen-3-ol and (Z)-4-heptenal have been considered to play an important role in the formation of mushroom aroma [18]. Moreover, according to our findings, the newly formed compound 1-octen-3-one was one of the most important contributors to the formation of the unique mushroom flavor of FMT, which has been rarely noticed in previous studies, and 1-octen-3-one also contributed to the formation of herbal and earthy flavors. After fermentation, “aldehydic”, “nut”, and “waxy” were no longer the dominant aroma attributes of the tea sample, which may be related to the decrease in the contents of heptanal, nonanal, octanal, and 6-methyl-5-hepten-2-one or the decrease of ROAV of some aroma compounds. According to KEGG analysis and previous research, phenylalanine metabolism, monoterpenoid biosynthesis, and fatty acid metabolism may have contributed to the biotransformation of the key aroma-active compounds during the artificial fermentation by *E. cristatum* (Figure 6) [14,30,36,37,38]. Among them, fatty acid metabolism might play a crucial role in the biotransformation of flavor substances.

## 4. Conclusions

In this study, we evaluated the sensory properties, including chromaticity, taste properties, and flavor characteristics, of MT and FMT. After fermentation, the color of the tea infusion became reddish-brown, and the taste property improved. The composition of the VOCs in MT was significantly changed after fermentation. 1-octen-3-one, (E)-2-nonenal, trimethyl-pyrazine, 2-pentylfuran, and heptanal were the potential aroma markers between MT and FMT. Mushroom, herbal, and earthy were the unique dominant flavor attributes of FMT, and we found that 1-octen-3-one was one of the most important aroma compounds in FMT, which has rarely been reported in previous studies of tea. This study provided a theoretical basis for the improvement in the sensory qualities of fermented mulberry leaf tea and is helpful for the popularization of this product. It has reference value for further improvements in the sensory qualities of fermented mulberry leaf tea in actual production. The methods and results of our study may serve as a reference for the sensory quality evaluations of other fermented products.

## Figures and Tables

**Table 1:** Key aroma-active compounds (ROAV ≥ 1) in MT and FMT and their flavor descriptors.

Compounds	Class	CAS	Range of Odor Min	Range of Odor Max	ROAV of MT	ROAV of FMT	Flavor Description	Fold Change (FMT vs. MT)	LRI
2-ethylhexanol	alcohols	104-76-7	0.198	-	269.16	316.75	green, rose, fresh, floral, sweet, citrus	2.47	993
1-pentanol	alcohols	71-41-0	5.5	305,000	7.31	1.6	balsamic, vanilla, sweet, oil	0.43	901
1-octanol	alcohols	111-87-5	0.9	1690	1.41	1.13	green, rose, mushroom, orange, aldehydic, waxy	0.94	1004
1-octen-3-ol	alcohols	3391-86-4	11	-	-	8.03	green, mushroom, fungal, earthy	+∞	1060
heptanal	aldehydes	111-71-7	0.003	-	2878.87	538.62	citrus, fatty, herbal, aldehydic, green, fresh,	0.38	1030
(E)-2-octenal	aldehydes	2548-87-0	0.003	-	526.88	729.36	herbal, green, fresh, nut, fatty, waxy	2.81	1294
pentanal	aldehydes	110-62-3	0.4	4970	86.18	-	fruity, malt, nutty, almond, berry	0	1404
benzeneacetaldehyde	aldehydes	122-78-1	1	-	39.84	20.53	honey, grapefruit, green, cocoa, floral, sweet, peanut, hyacinth	1.05	700
nonanal	aldehydes	124-19-6	1	-	19.93	8.81	grapefruit, fat, rose, green, fresh, aldehydic, citrus, fatty, orange peel, waxy	0.88	962
octanal	aldehydes	124-13-0	2.5	-	2.64	-	green, aldehydic, lemon, citrus, fatty, orange peel, waxy	0.71	1045
hexanal	aldehydes	66-25-1	20	-	1.78	-	fruity, sweaty, grass, fatty, aldehydic, green, fresh	0.52	1066
(E)-2-nonenal	aldehydes	18829-56-6	0.0002	-	-	1756.96	fatty, cucumber, green	+∞	1123
vanillin	benzenoids	121-33-5	0.0002	92.9	100	-	vanilla, creamy, chocolate, sweet	0	1104
benzaldehyde	benzenoids	100-52-7	1.5	783,000	79.1	27.49	almond, bitter, sweet, cherry, fruit, vanilla	0.7	891
naphthalene	benzenoids	91-20-3	1.9	1020	2.97	5.04	pungent, tar, mothballs	3.5	937
phenol	benzenoids	108-95-2	4.5	1950	2.34	3.1	plastic, rubber, phenol, phenolic	2.7	765
ethyl 2-methylbutyrate	esters	7452-79-1	1.6	-	1.33	-	fruity, green, apple, sweet	0	703
methyl salicylate	esters	119-36-8	40	-	-	4.44	mint, wintergreen, caramel	65.61	1182
2-pentylfuran	heterocyclic compounds	3777-69-3	0.006	-	10,601.94	812.71	fruity, green, earthy, vegetable, butter	0.15	1003
trimethyl-pyrazine	heterocyclic compounds	14667-55-1	0.023	-	1311.26	3026.34	nutty, cocoa, hazelnut, roasted, peanut, earthy,	4.64	981
2-ethylfuran	heterocyclic compounds	3208-16-0	2.3	-	5.06	-	earthy, burnt, malty, sweet, coffee-Like	0	1189
pyridine	heterocyclic compounds	110-86-1	10	12,000	-	6.02	putrid, fishy, sour, burnt	+∞	801
2-methylpyridine	heterocyclic compounds	109-06-8	2.6	23.6	-	3.34	bitter, sweat	+∞	1070
2-undecanone	ketones	112-12-9	0.0044	-	198.64	85.63	fruity, green, fresh, orange, floral, fatty, waxy	0.87	849
acetophenone	ketones	98-86-2	0.24	590	27.23	299.39	flower, must, hawthorn, almond, sweet, oranges	22.13	986
isophorone	ketones	78-59-1	0.3	190	22.51	118.06	fruity, green, cedarwood, sweet, camphoraceous, woody	9.93	1162
2-heptanone	ketones	110-43-0	0.75	710	19.85	3.98	herbal, fruity, sweet, mushroom, woody	0.41	1273
6-methyl-5-hepten-2-one	ketones	110-93-0	50	-	1.11	-	green, nutty, hazelnut, mushroom, bitter, apple, lemongrass, citrus	0.66	980
1-octen-3-one	ketones	4312-99-6	0.005	-	-	3950.26	herbal, mushroom, earthy	+∞	979
damascenone	ketones	23696-85-7	0.1	-	-	215.69	sweet, rose	+∞	0
a-pinene	lipids and lipid-like molecules	80-56-8	0.06	19,000	8.13	4.54	turpentine, fresh, minty, terpene, sweet, camphor, earthy, woody	1.12	746
a-terpineol	lipids and lipid-like molecules	98-55-5	1	-	2.01	4.47	piney, iris, teil	4.49	1192
citral	lipids and lipid-like molecules	5392-40-5	0.024	32	-	322.33	lemon, flowery, citrous	+∞	816

-: not detected. FC: fold change. LRI: linear retention index.

## Data Availability

The original contributions presented in the study are included in the article/Supplementary Material, further inquiries can be directed to the corresponding authors.

## Supplementary Materials

The following supporting information can be downloaded at https://www.mdpi.com/article/10.3390/foods13152347/s1, Figure S1. Three-dimensional total ion flow chromatogram of MT (A) and FMT (B).
