# Supplementary material for: Effects of Fermentation with Eurotium cristatum on Sensory Properties and Flavor Compounds of Mulberry Leaf Tea
Source: Foods. 2024 Jul 25;13(15):2347. doi: 10.3390/foods13152347 (PMC11311662; doi:10.3390/foods13152347)
Supplement: Supplementary file 1 [file foods-13-02347-s001.zip › foods-3094449-supplementary.pdf]

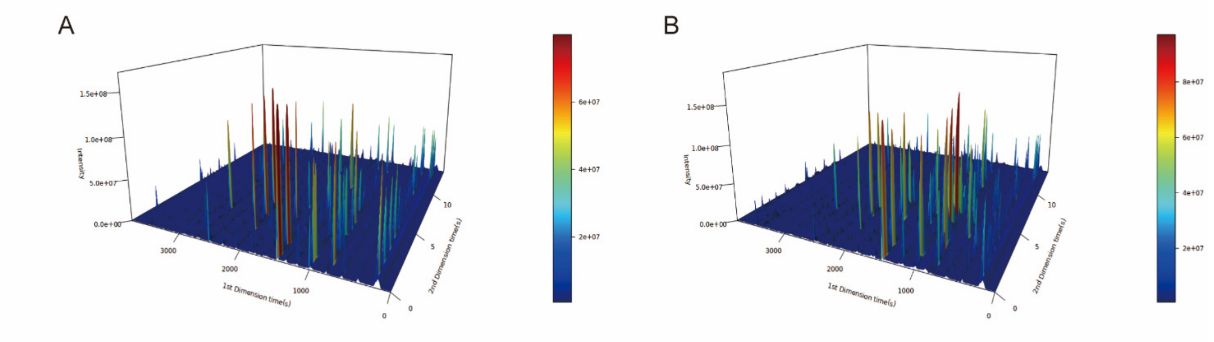

Figure S1. Three-dimensional total ion flow chromatogram of MT (A) and FMT (B)
The horizontal coordinate is one-dimensional retention time (s), and the vertical coordinate is two-dimensional retention time (s). Color and peak height indicate the ionic response strength. The redder the color, the stronger the response.
